# IMD-0354 Targets Breast Cancer Stem Cells: A Novel Approach for an Adjuvant to Chemotherapy to Prevent Multidrug Resistance in a Murine Model

**DOI:** 10.1371/journal.pone.0073607

**Published:** 2013-08-27

**Authors:** Azucena Gomez-Cabrero, Wolfgang Wrasidlo, Ralph A. Reisfeld

**Affiliations:** 1 Department of Immunology and Microbial Sciences, The Scripps Research Institute, La Jolla, California, United States of America; 2 Moores Cancer Center, University of California San Diego, San Diego, California, United States of America; University of Pécs Medical School, Hungary

## Abstract

Although early detection of breast cancer improved in recent years, prognosis of patients with late stage breast cancer remains poor, mostly due to development of multidrug resistance (MDR) followed by tumor recurrence. Cancer stem cells (CSCs), with higher drug efflux capability and other stem cell-like properties, are concentrated in a side population (SP) of cells, which were proposed to be responsible for MDR and tumor repopulation that cause patients to succumb to breast cancer. Therefore, targeting of CSCs as an adjuvant to chemotherapy should be able to provide a more effective treatment of this disease. Here, we used IMD-0354, an inhibitor of NF-κB, identified for targeting CSCs, in a combination therapy with doxorubicin encapsulated in targeted nanoparticles. IMD-0354 did target CSCs, evidenced by a decrease in the SP, demonstrated by the inhibition of the following: dye/drug efflux, reduction in ABC transporters as well as in colony formation in soft agar and low attachment plates. Decrease of stem-like gene expression of Oct4, Nanog and Sox2, and apoptosis resistance related to the Survivin gene also was observed after treatment with this compound. In addition, IMD-0354 targeted non-CSCs as indicated by reducing viability and increasing apoptosis. Targeted drug delivery, achieved with a legumain inhibitor, proved to enhance drug delivery under hypoxia, a hallmark of the tumor microenvironment, but not under normoxia. Together, this allowed a safe, non-toxic delivery of both anticancer agents to the tumor microenvironment of mice bearing syngeneic metastatic breast cancer. Targeting both bulk tumor cells with a chemotherapeutic agent and CSCs with IMD-0354 should be able to reduce MDR. This could eventually result in decreasing tumor recurrences and/or improve the outcome of metastatic disease.

## Introduction

Breast cancer is the second most commonly diagnosed malignancy among American women, and is second in cancer related deaths [1]. Although death rates from breast cancer decreased modestly over the last few years, more efficient therapies are urgently needed, especially in the case of aggressively invading breast cancer. Alternatively, treatment of breast cancer with radio- and/or chemotherapy frequently leads to multiple drug resistance (MDR) and tumor recurrence.

Doxorubicin (Dox) is a chemotherapeutic drug commonly used to treat breast cancer. However, its side effects, particularly cardiac toxicity, make it a poor option for cancer treatment [2]. In recent years, encapsulation of Dox into liposomal nanoparticles, considerably reduced heart toxicity. However, nonspecificity of these lipid nanoparticles does not reduce toxicity in other organs [3]. Therefore, major improvements are required for the safe and effective chemotherapy of breast and other solid tumors.

A number of studies identified subpopulations of cells within tumors that drive their growth and recurrence, designated cancer stem cells (CSCs) [4]. CSCs are small subpopulations of cells with some stem cell-like properties, such as self-renewal, colony formation, expression of stem cell genes, and ability to repopulate the tumor mass. In addition, CSCs overexpress multidrug transporters of the ATP-binding cassette (ABC) family, such as ABCG2 and MDR1, and greatly contribute to the typical MDR of such cells [5]. Together, these observations suggest that many cancer therapies, while killing the bulk of tumor cells, may ultimately fail because they do not eliminate CSCs, which survive to regenerate new tumors. The presence of CSCs in cancer cell populations is measured operationally *in vivo*, based on their ability to seed tumors at limiting dilutions. CSC-enriched cancer cell populations also exhibit characteristic properties *in vitro*. Thus, CSC-enriched subpopulations can be isolated based on characteristic cell-surface marker profiles [6,7]. In breast cancer, CSCs were reported enriched in the CD44high/CD24low subfraction of cells [8]. Further, CSC-enriched populations formed spherical colonies in suspension cultures, termed tumor mammospheres [9] or tumor-spheres. Finally, populations enriched in CSC also exhibit increased resistance to chemotherapeutic agents and ionizing radiation [10–12].

In principle, automated screening technologies should facilitate the identification of agents that kill CSCs. However, since CSCs generally comprise only small minorities within cancer cell populations, standard high-throughput cell viability assays applied to bulk populations of cancer cells fail to identify agents with CSC-specific toxicity. Accordingly, effective screening for agents that preferentially kill CSCs depends on their ability to propagate stable, highly enriched populations of CSCs *in vitro*. However, this is currently not possible for CSCs of solid tumors, as enrichment of breast CSCs is rapidly diminished during *in vitro* culture [13]. A better approach would be to apply screening to the whole population of cancer cells (CSCs and bulk cells), and analyze the resulting toxicity only on cells identified as CSCs. Among the different characteristics of CSCs, surface markers would be a first option for high-throughput screening. Unfortunately, surface markers for CSCs are very heterogeneous [14], making their use for high-throughput screening difficult. Instead, a functional assay of ‘stemness’ should have a more general applicability for different patients, cell lines and cancers.

Here, we adapted such a functional cell-based assay to identify CSCs for a high-throughput screening platform. Since CSCs overexpress ABC transporters, they can efflux dyes and other compounds in the same way as chemotherapeutic agents. Therefore, CSCs readily efflux Hoechst 33342 dye and appear as a subpopulation of cells, called side population (SP), with low staining for this dye compared to the bulk of tumor cells. Interestingly, IMD-0354 was one of the hit compounds we found that reduced the side population, or CSCs. IMD-0354, an indirect inhibitor of NF-κB, was first described as cardio-protective in ischemia/reperfusion injury [15]. Although, this inhibitor is usually studied as an anti-inflammatory agent, some studies pointed to its application to treat cancer [16]. In addition to inflammation, the NF-κB signaling pathway has been linked to proliferation, anti-apoptosis, angiogenesis and immune tolerance in the tumor microenvironment. This makes the NF-κB pathway an attractive target for cancer treatment. NF-κB is constitutively active in many cancer cells and resides in the nucleus. However, this activation is due to chronic stimulation in the IKK pathway, or other defective genes encoding IkBa [17,18].

We recently developed a novel nanoparticle (NP) targeting strategy utilizing a ligand, R11-a, that will bind covalently to legumain to target the tumor microenvironment (TME) [19]. Legumain is the only mammalian asparaginyl endopeptidase, which is overexpressed on tumor cells under hypoxic stress [20], a hallmark of solid tumors also expressed on tumor-associated macrophages (TAMs) [21–23]. Although tumor hypoxia is present in all solid tumors, it is a poor prognostic factor for patient outcome [24,25], especially since it is likely a cause of hypoxic induction of angiogenic factors, chemokines, oncogenes, and other drivers of tumor progression [26] that confer metastatic competence [27]. Significantly, legumain expression was reported to increase cell migration and invasion *in vitro*, and to correlate with poor prognosis and malignancy [28,29]. This enzyme’s vesicular staining pattern is associated with more invasive and aggressive prostate cancer [30]; and legumain activated prodrugs suppress tumor growth and metastasis without toxicity [31]. In fact, we previously demonstrated that legumain-targeting dramatically improved NP drug delivery to solid tumors, while preventing non-specific accumulation in the reticuloendothelial system [20].

Here, we hypothesize that MDR can be overcome by sensitizing CSCs to chemotherapy with targeted molecular therapeutics. Using our functional assay focused on CSCs’ ability to efflux lipophilic dyes, hit compounds were rigorously characterized *in vitro* and loaded, together with chemotherapeutic agent such as Dox, into ligand-targeted nanoparticles (tNPs). This strategy was previously established in our laboratory, as highly effective for payload delivery to the tumor microenvironment. In addition, xenografts in syngeneic mouse models served to evaluate the efficacy of such targeted multi-payload NPs to prevent MDR, spontaneous metastasis and cancer recurrence.

Here, we describe the effects of the NF-κB inhibitor, IMD-0354, on CSCs of human and murine breast cancer cells and its potential adjuvant properties to enhance chemotherapeutic tumor cytotoxicity. For *in vivo* delivery to the TME, we developed a novel loading strategy to encapsulate IMD-0354 into legumain-targeted NPs. Finally, we demonstrated that this novel combination therapy with Dox and IMD-0354 improved the anti-tumor effects of Dox, with the potential perspective of enhancing protection against breast cancer recurrence.

## Materials and Methods

### Cells and drugs

The 4T1 murine breast carcinoma cell line was provided by Suzanne Ostrand-Rosenberg (University of Maryland) [32]. The FL4T1 firefly luciferase expressing murine breast carcinoma cell line was provided by Dr. Brunhilde Felding-Habermann (The Scripps Research Institute) [33]. MDA-MB-231 cells were purchased from ATCC. All cells were maintained in RPMI-1640 medium (Gibco), supplemented with 10% FBS, 1% HEPES, 1% sodium bicarbonate and 1% sodium pyruvate. The 4T1 cell line was authenticated by *in vivo* growth/metastasis in BALB/c mice, via expression of IL-6 and S100A8/A9, and resistance to 6-thioguanine. Cells were tested for negativity of mycoplasma by the in-house Center for Antibody Development and Production core.

IMD-0354 was purchased from Tocris Cookson Inc.; Dox from LC Laboratories; and CoCl2, mitoxantrone and cisplatin from Sigma-Aldrich.

### Cell viability assay (MTT)

For cell viability assays with MTT, cells were incubated with 0.5 mg/ml thiazolyl blue tetrazolium bromide (Alfa Aesar) for 10-60 min; the formazan product was then solubilized in ethanol; and its OD at 570 nm measured by a plate reader spectrophotometer.

### Flow cytometry

For analysis of the side population (SP) phenotype, cells were stained with 1 mg/ml of Hoechst 33342 dye for 1 h in DMEM medium containing 2% FBS and 10 mM HEPES; cells were then washed twice with PBS and detached with CellStripper (Cellgro) for 15 min. and collected with HBSS containing 2% FBS, 10 mM HEPES and 0.625 µg/ml propidium iodide (PI). Hoechst dye was excited at 407 nm by a trigon violet laser and its dual wavelengths were detected using 450/40 (Hoechst 33342-Blue) and 695/40 (Hoechst 33342-Red) filters. PI was excited at 488 nm using an octagon blue laser and fluorescence detected by a 675/20 filter. Dead cells were excluded by gating on forward and side scatter and by eliminating PI-positive populations.

Apoptosis was analyzed with Aposcreen Annexin V-FITC following manufacturer’s instructions (SouthernBiotech).

For analysis of legumain expression, immunocytochemistry was performed with anti-legumain antibody (R&D Systems) and anti-sheep-FITC (Southernbiotech).

Cells were analyzed by LSR-II FACS (Becton Dickinson) and data analyzed with FlowJo software (Tree Star, Inc.).

### Colony formation

For 3D colony formation, cells were plated as 10,000 cells/well in 6-well ultra-low attachment plates, and cultured with MDEM/F12 (1:1) medium with L-glutamine and HEPES, supplemented with 1x B27, 20 nm/ml FGF and 20 ng/ml bFGF. Every day, the drug or vehicle was added to a final concentration of 1 µM. Every three days, fresh medium was added. Colonies were counted as groups of three or more cells.

For soft agar colony formation, cells were plated as 5,000 cells/well in 0.35% agar in culture medium in 6-well culture plates, previously coated with 0.5% agar in culture medium, and incubated for several days. Finally, plates were stained with crystal violet for colony counting.

### Western blots

Protein extracts were prepared as previously described [34]. Whole extracts were obtained with RIPA buffer; cytosolic and nuclear extract were prepared as described prior [35]. Western blots were done as previously described [34,36] and probed with the following antibodies: beta-actin, MDR1, ABCG2, Nanog, Oct4, Sox2, Survivin (all Santa Cruz Biotechnology).

### Nanoparticle formulation

Synthesis of the legumain-specific inhibitor RR-11a was previously described [37]. For conjugation to lipids, we modified the ligand RR11a by introducing a NHS ester group for coupling to the amino group of DOPE. Phospholipids (Avanti Polar Lipids) were dissolved in chloroform. RR-11a was conjugated to 1,2-dioleoyl-sn-glycero-3-phosphoethanolamine (DOPE) using triethylamine as a catalyst. The resulting compound was combined with 1,2-dioleoyl-sn-glycero-3-phosphocholine (DOPC), DOPE, cholesterol, and 1,2-dioleoyl-sn-glycero-3-phospho- ethanolamine-N-[methoxy(polyethylene glycol)-2000], DOPE- PEG, at molar ratios of 1.1:6.7:6.7: 2.2:1, as previously described [38]. Dox was loaded into nanoparticles as previously described [19]. IMD-0354 was dissolved in 10% BSA in PBS pH 7.4, and lipids were dissolved in chloroform and the solvent evaporated to leave a lipid film. IMD-0354/BSA solution was added to this film and the mixture treated as for Dox nanoparticles. The non-encapsulated IMD-0354/BSA solution was removed by separation using sepharose(R) CL-4B (Sigma-Aldrich). All nanoparticles were filter sterilized.

### Animal experiments

Female BALB/c mice were purchased from The Scripps Research Institute Rodent Breeding Facility and housed in an AAALAC accredited facility. Mice were injected intravenously with 5x10^5^ FL4T1 cells in 100 µl PBS on day 0. Next day, seeding of bioluminescent cancer cells on internal organs was confirmed by *in vivo* imaging after intraperitoneal injection of firefly D-luciferin potassium salt (Biosynth) in PBS. Mice received 6 intravenous injections, at 3-day intervals, of tNP-Dox, tNP-IMD-0354, or tNP-Dox-IMD-0354 in 200 µl PBS. Tumor burden and progression were followed by *in vivo* bioluminescence imaging, and body weight was annotated periodically. Mice were sacrificed 24 hours after the final treatment and body weight determined.

### Ethics statement

All animal procedures were performed in strict accordance with the recommendations in the Guide of the Care and Use of Laboratory Animals of the National Institutes of Health. The protocol was approved by the Institute Animal Care and Use Committee of The Scripps Research Institute (animal protocol # 08-0148). All efforts were made to minimize suffering.

### Statistical analysis

The statistical significance of differential findings between experimental groups and controls was determined by 2-tailed Student’s t test using Prism software (GraphPad). Results were regarded as significant if P < 0.05.

## Results

### Both human and murine breast cancer cells are resistant to common chemotherapeutic agents

**Figure 1:**
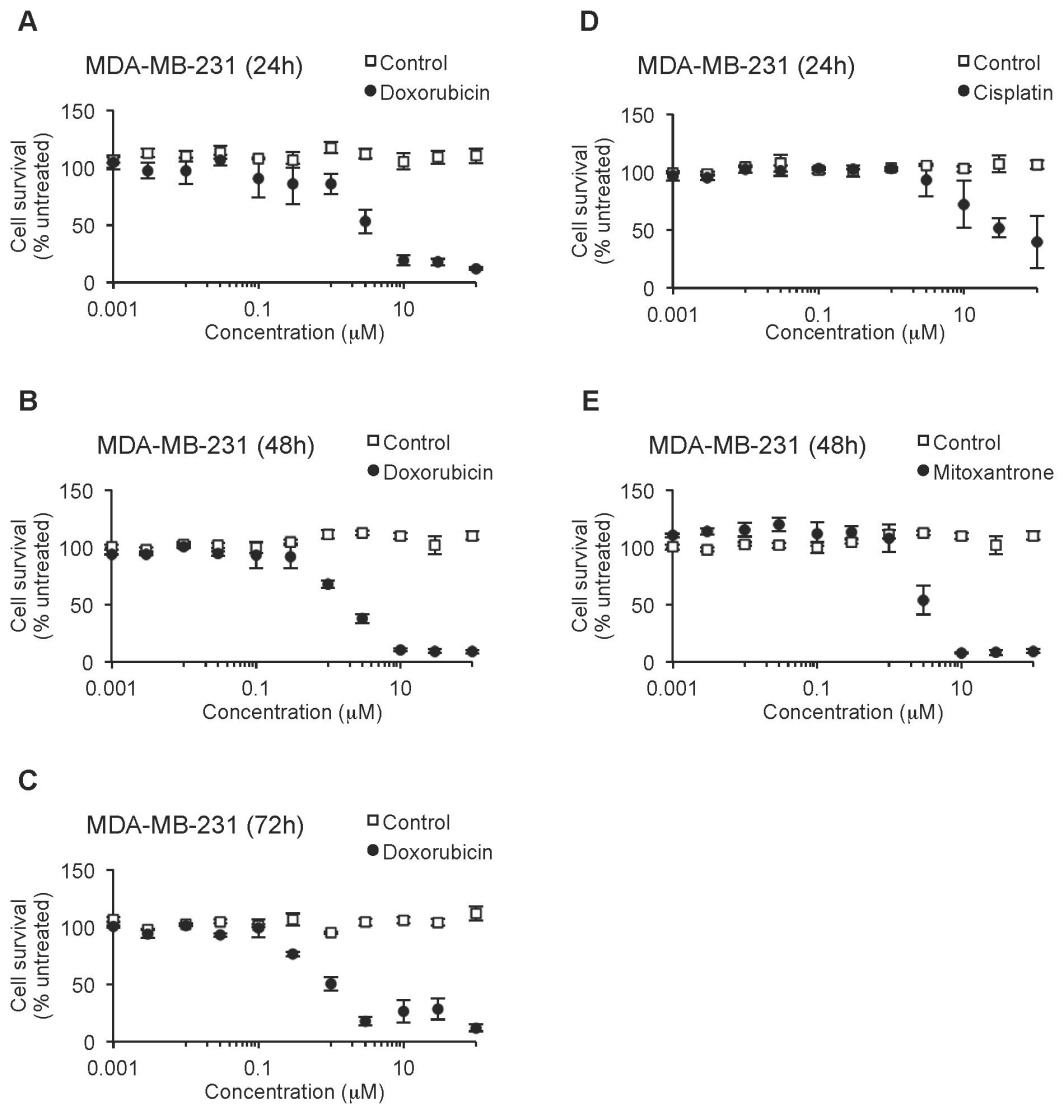
Human breast cancer cells are chemoresistant to different chemotherapeutic agents. Viability of MDA-MB-231 cells was assessed by MTT assay after treatment with doxorubicin for 24 h (A), 48 h (B) and 72 h (C), or cisplatin for 24 h (D), or mitoxantrone for 48 h (E). Data are shown as mean ± SEM.

MDA-MB-231 is a cell line derived from highly malignant and metastatic human breast carcinoma [39], which is able to reproduce tumor growth and metastasis in immunosuppressed mice similar to tumor progression in patients [40,41]. MDA-MB-231 cells were treated with Dox at different concentrations for 24 to 72 hours, and their viability analyzed by the cell proliferation/cytotoxicity assay MTT (Figure 1A–C). In these cells, Dox showed an IC_50_ (inhibitory concentration with a 50% cytotoxic effect) in the micromolar range (IC_50_ = 5.1 µM at 24 h; 3.9 µM at 48 h; and 1.5 µM at 72 h), with typical cellular death and apoptotic morphology, which appeared under the microscope proportionally to cytotoxic effect. Similar results were obtained when these cells were treated with mitoxantrone (Figure 1E; IC_50_ = 5.3 µM at 48 h). In the case of cisplatin, MDA-MB-231 cells were more resistant to this chemotherapeutic drug than to the other two drugs, with an IC_50_ close to 100 µM (Figure 1D; IC_50_ = 67.3 µM at 24 h). Theses results showed that MDA-MB-231 cells have a very low sensitivity to these three common chemotherapeutics.

**Figure 2:**
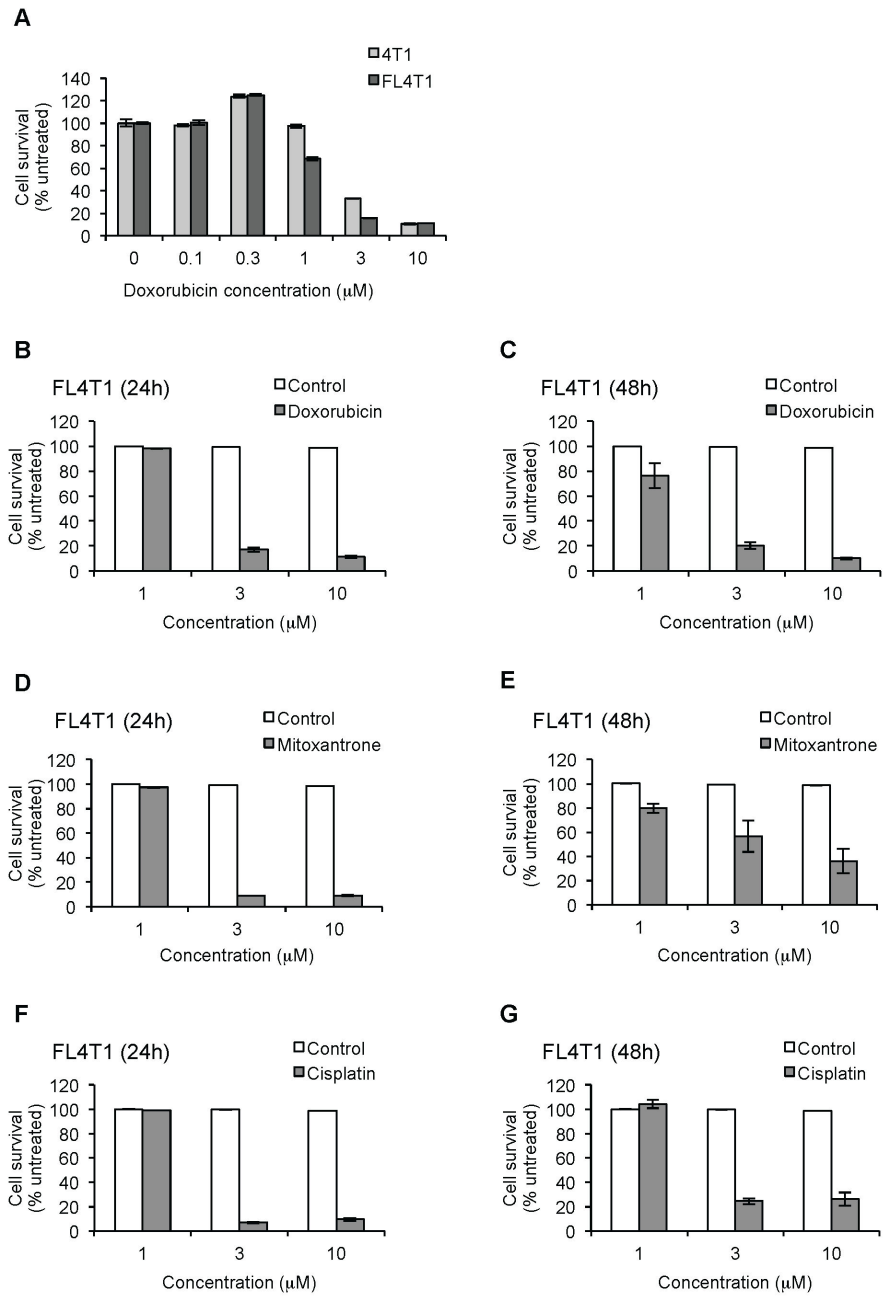
Murine breast cancer cells are chemoresistant to different chemotherapeutic agents. Viability of 4T1 or FL4T1 cells was assessed by the MTT assay after treatment with: doxorubicin for 48 h comparing both cell lines (A); doxorubicin for 24 (B) and 48 h (C); mitoxantrone for 24 (D) and 48 h (E); cisplatin for 24 (F) or 48 h (G). Data are shown as mean ± SEM.

The FL4T1 cell line is a firefly luciferase expression clone derived from 4T1 cells. The 4T1 cells are derived from a murine breast cancer cell line able to produce tumors and metastasis in syngeneic mouse models in a similar way as that of human breast cancer [42,43]. FL4T1 cells showed a similar sensitivity to Dox at 48 hours than parental 4T1 cells as shown by MTT assay (Figure 2A; IC_50_ = 2.0 and 2.5 µM, respectively). FL4T1 cells treated with Dox for 24 or 48 h showed resistance to Dox with an IC_50_ in the micromolar range (IC_50_ = 2.2 and 1.9 µM, respectively) and apoptotic cell morphology, similarly to the human breast cancer cell line MDA-MB-231 (Figure 2B and C). In addition, FL4T1 revealed a similar resistance to mitoxantrone (Figure 2D and E) and cisplatin (Figure 2F and G) than the human counterpart MDA-MB-231 (IC_50_ = 2.1 µM at 24 h; and 6.4 µM at 48 h for mitoxantrone; IC_50_ = 2.1 µM at 24 h; and 2.4 µM at 48 h for cisplatin). All together, these results indicated a chemoresistant behavior for both human and murine breast cancer cell lines, similar to the multidrug resistance affecting most breast cancer patients.

### Human and murine cancer cells contain a subpopulation of CSCs

**Figure 3:**
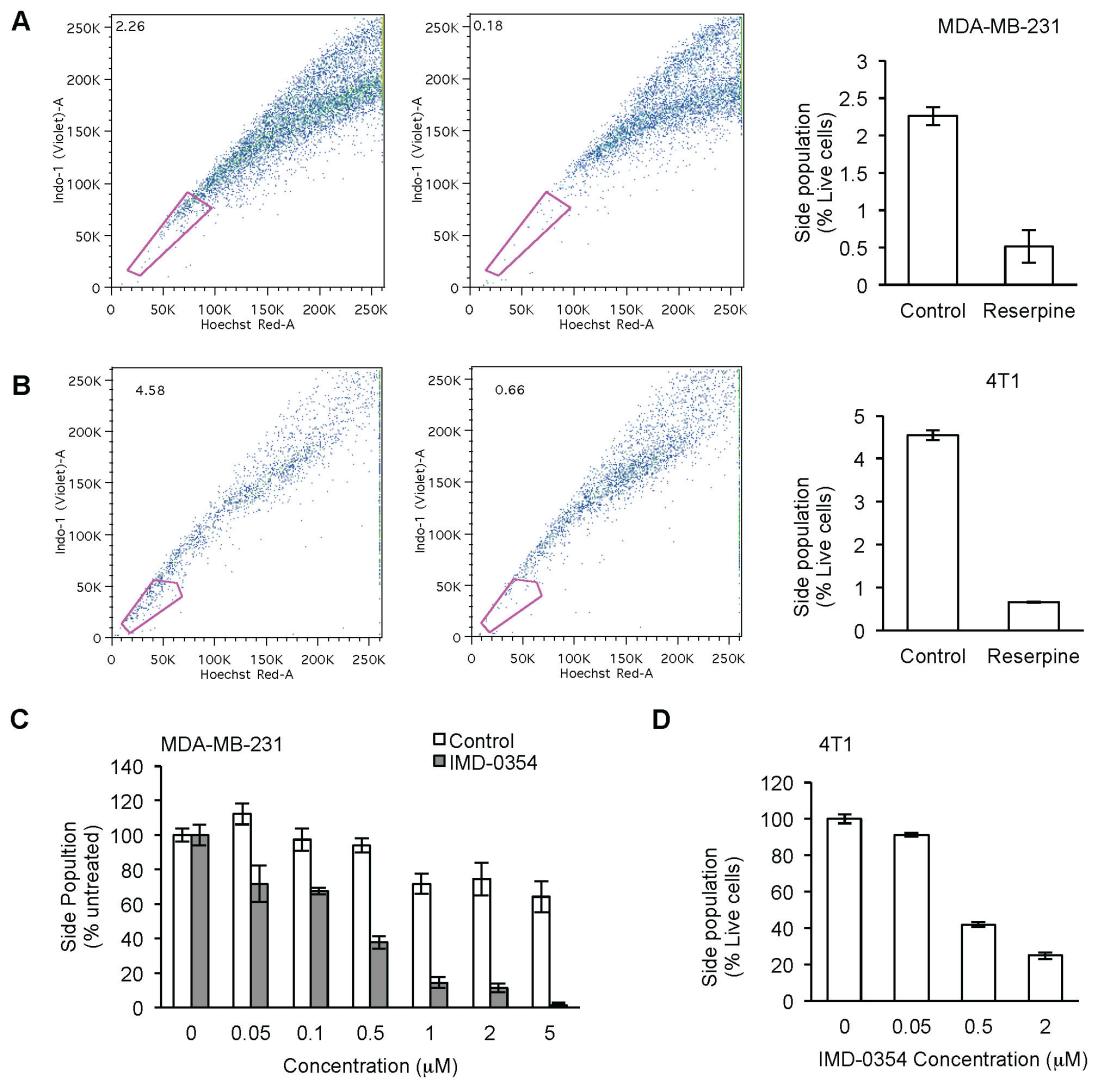
IMD-0354 inhibits side population of CSCs in human and murine breast cancer cells. Side population of CSCs as analyzed by FACS and their Hoechst 33342 dye efflux inhibition by reserpine in MDA-MB-231 (A) and 4T1 (B). Dose curve of IMD-0354 on side population in MDA-MB-231 (C) and 4T1 (D). Data are shown as mean ± SEM.

Different tests can be applied to prove the presence of CSCs in human MDA-MB-231 and murine 4T1 breast cancer cells. A fast, marker-independent way to identify CSCs is to stain cells with Hoechst 33342 dye and then analyze their red and blue/violet fluorescence by FACS. CSCs overexpress different drug efflux pumps and are able to exclude the dye, appearing as a SP with low fluorescence for this dye. However, non-CSCs or bulk tumor cells are unable to efflux the dye as efficiently as CSCs and therefore appear as a non-SP with higher dye fluorescence. SP can be identified as the population that disappears when a drug efflux pump inhibitor is used during staining, such as reserpine or verapamil. Reserpine and other pump inhibitors reduce the ability of CSCs to efflux dye to an extent similar to non-CSCs [44,45]. Human MDA-MB-231 breast cancer cells presented distinct SP of cells (Figure 3A; 2.06 ± 0.21% cells when compared to 0.27 ± 0.12% cells in reserpine treated cells); 4T1 cells also showed a SP of CSCs (Figure 3B; 4.543 ± 0.114% cells compared to 0.66 ± 0.012% cells in reserpine treated cells). These results are in agreement with previous findings by others and ourselves [46,47].

### In vitro effects of IMD0354 on breast CSCs

IMD-0354, an inhibitor of the NF-κB pathway, was initially designed to inhibit inflammation [48,49], but recently proven to have anticancer properties [16,50,51]. Here, we wanted to assess if IMD-0354 also affects CSCs, as indicated by prior screening. MDA-MB-231 cells, when treated with IMD-0354, showed a decrease in SP of CSCs in a dose dependent manner (Figure 3C), with an IC_50_ in the nanomolar range (378 nM). Similarly, IMD-0354 decreased SP in 4T1 cells in a concentration dependent manner, with an IC_50_ also in the nanomolar range (Figure 3D; 427 nM).

**Figure 4:**
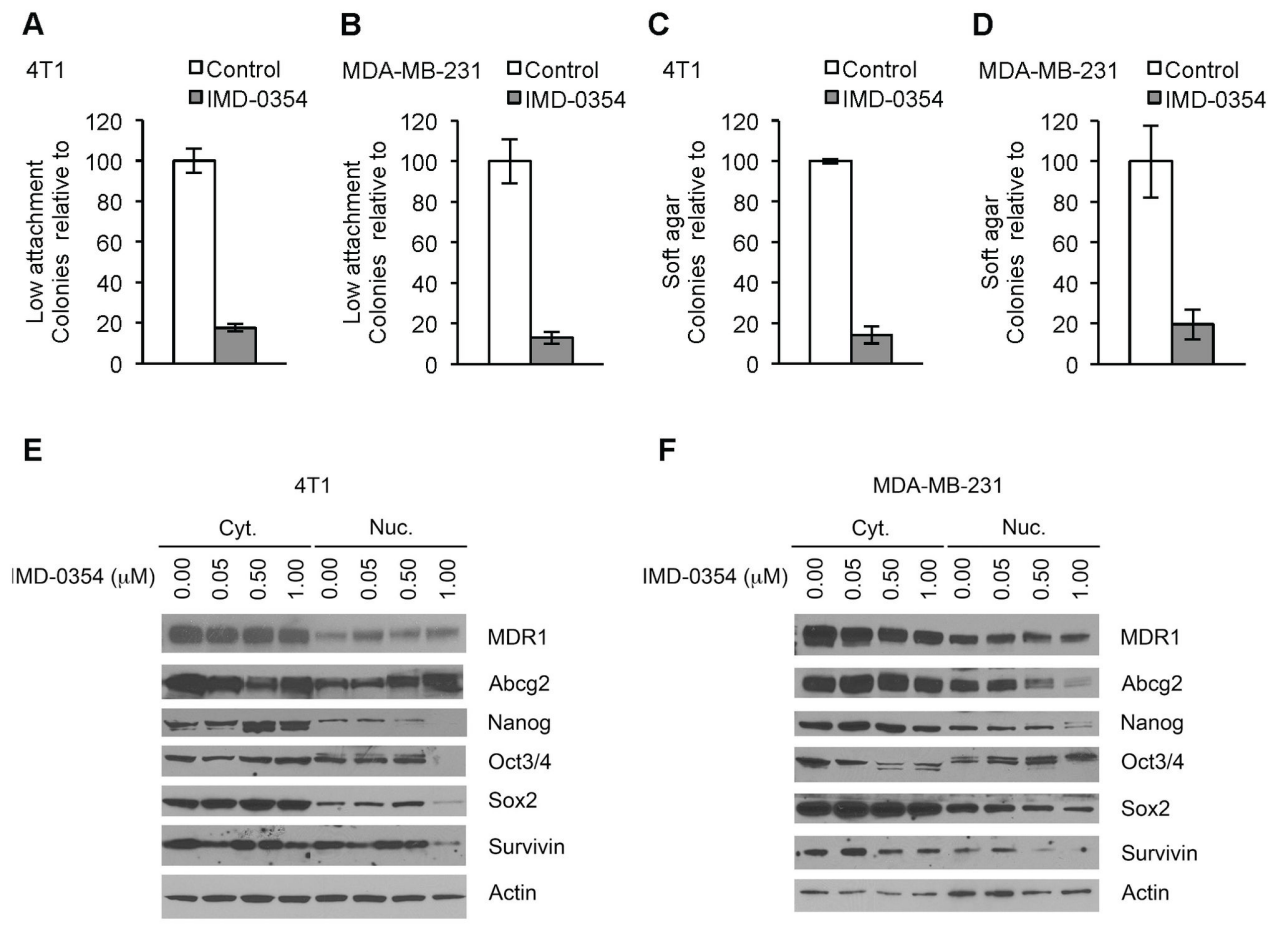
Effects of IMD-0354 on CSCs. Number of colony formation in low attachment plates of 4T1 (A) and MDA-MB-231 (B) treated with 1 µM IMD-0354. Number of colony formation in soft agar of 4T1 (C) and MDA-MB-231 (D) treated with 1 µM IMD-0354. Western blot of CSCs gene expression of cytosolic and nuclear extract from 4T1 (F) and MDA-MB-231 (G) cells treated with IMD-0354. Data are shown as mean ± SEM.

Breast CSCs express other stem-like properties, including the ability to grow as 3D spheres or colonies under certain conditions, such as low attachment plates and soft agarose medium [52]. When murine 4T1 breast cancer cells were grown on low attachment plates, they were able to form spheres of several cells (colonies); however, 1 µM IMD-0354 inhibited 4T1 cells’ ability to form such tumor-spheres in low attachment plates (Figure 4A; 17.6 ± 1.9% colonies compared to control). Similarly, human breast cancer cells MDA-MB-231 treated with 1 µM IMD-0354 produced fewer colonies than controls in low attachment plates (Figure 4B; 12.9 ± 2.8% colonies compared to control). Additionally, 1 µM IMD-0354 was able to reduce the number of colonies formed by 4T1 cells in soft agarose (Figure 4C; 14.1 ± 4.3% colonies compared to control) and this effect could be reproduced in human breast cancer cells MDA-MB-231 (Figure 4D; 19.6 ± 7.3% colonies compared to control).

Another CSC trait is their stem-like gene expression profile, including ABC transporters MDR1 and ABCG2, stemness genes Nanog, Oct4 and Sox2, and the antiapoptotic gene Survivin [47,52–55]. The expression of these genes was assessed at the protein level by Western blots of cytosolic (cyt.) and nuclear (nuc.) extracts. 4T1 cells treated with 1 µM IMD-0354 showed a marked decrease in transcription factors nanog, oct4, sox2 and survivin in the nuclear extract, indicating a reduction of activity of these proteins and their target transcripts (Figure 4E). Additionally, nanog, oct4 and sox2 proteins increased in the cytosolic fraction, indicating a reduction of these transcription factor activities due to subcellular localization. Protein expression of ABC transporters MDR1 and ABCG2 was slightly reduced by 1 µM IMD-0354 treatment when compared to controls in the cytosolic fraction, and inversely changed in the nuclear fraction. Furthermore, a similar result was observed in human MDA-MB-231 cells treated with IMD-0354 (Figure 4F): nanog, sox2 and survivin levels were reduced in the nuclear fraction; nanog and survivin levels were also increased in the cytosolic fraction; and mdr1 and abcg2 levels were slightly reduced in the cytosolic fraction. However, in IMD-0354 treated MDA-MB-231 cells, mdr1 and abcg2 levels in the nuclear fraction were reduced compared to controls; and oct4 levels decreased in the cytosolic fraction but increased in the nuclear fraction, compared to controls.

Taking together these results indicate that IMD-0354 reduced not only SP or dye efflux, but also CSCs subpopulations, including gene expression and tumor-sphere formation capability, in both human and murine breast cancer cell lines.

### In vitro effects of IMD0354 on breast cancer cells

**Figure 5:**
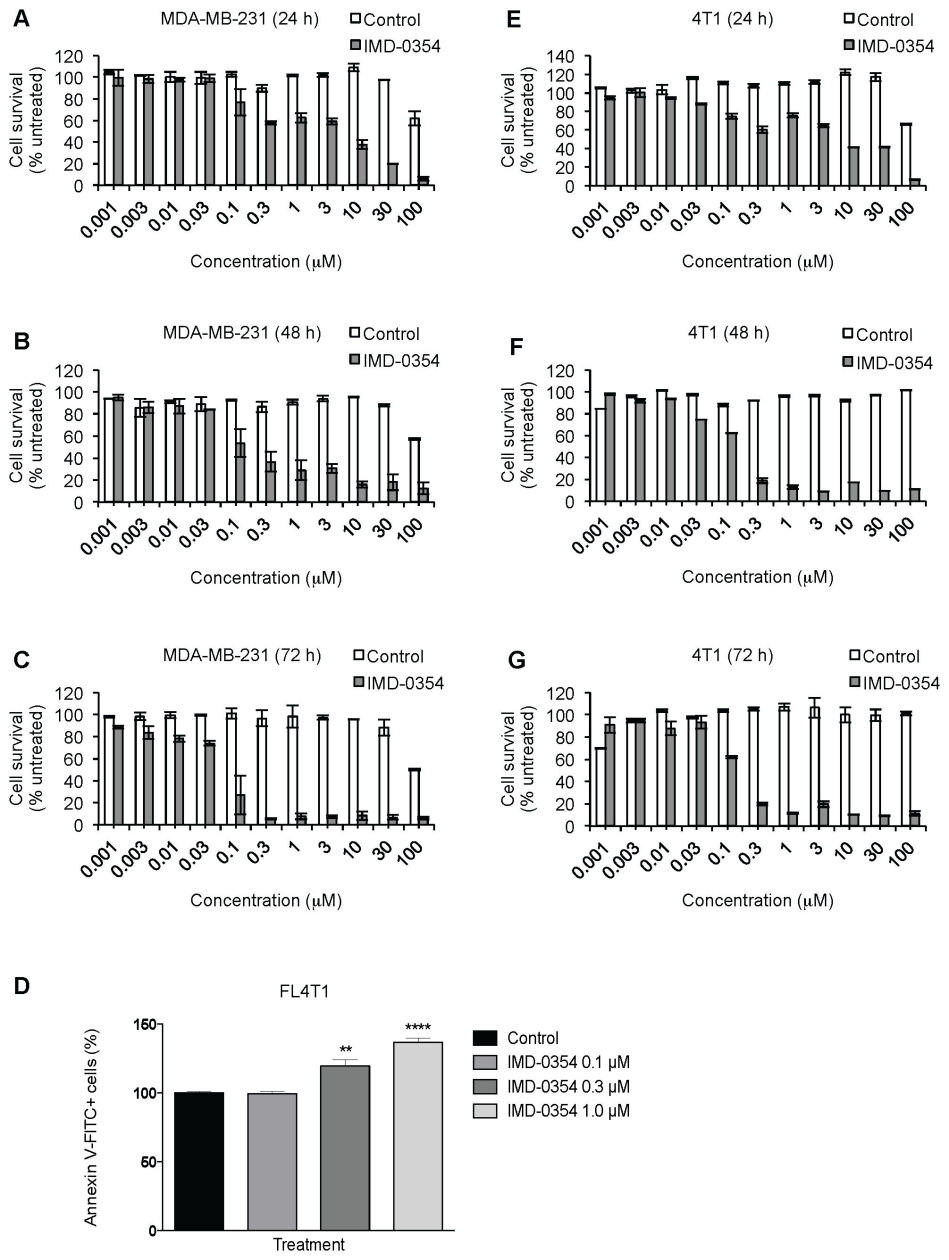
IMD-0354 effects on human and murine breast cancer cells. Viability of MDA-MB-231 cells was assessed by MTT assay after treatment with IMD-0354 for 24 h (A), 48 h (B) and 72 h (C). Apoptotic cells as Annexin V-FITC+ cells analyzed by FACS of FL4T1 cells treated with IMD-0354 (D). Viability of 4T1 cells was assessed by MTT assay after treatment with IMD-0354 for 24 h (E), 48 h (F) and 72 h (G). Data are shown as mean ± SEM. **P-value < 0.01; ****P-value < 0.001.

In addition to the IMD-0354 effects on CSCs, we wanted to know if this compound could also affect non-CSCs or bulk tumor cells. First, both human and murine breast cancer cells MDA-MB-231 and 4T1, respectively, were treated with 1 µM IMD-0354 and their viability assed by the MTT assay. Human MDA-MB-231 cells revealed low sensitivity to IMD-0354 at 24 h (IC_50_ = 0.34-5.79 µM), but a higher sensitivity to IMD-354 than to chemotherapeutic drugs such as Dox, mitoxantrone and cisplatin at 48 and 72 h, with an IC_50_ in the nanomolar range (Figure 5A–C; IC_50_ = 200 and 60 nM at 48 and 72 h, respectively). Similarly, 4T1 cells were initially resistant to IMD-0354 (IC_50_ = 7.5 µM), but showed a higher sensitivity to IMD-0354 than to chemotherapeutics at later time points (48 and 72 h), with an IC_50_ also in the nanomolar range (Figure 5E–G; IC_50_ = 160 and 160 nM at 48 and 72 h, respectively).

We then assessed whether the cytotoxic effect of IMD-0354 was due to an increase in apoptosis. To this end, murine FL4T1 cells treated with IMD-0354 were stained with Annexin V-FITC and analyzed by FACS to detect apoptotic cells (Figure 5D). IMD-0354 was able to increase apoptosis of Annexin V-FITC positive cells at 300 nM and 1 µM (19 and 37 % apoptosis increase, respectively, compared to control; P-value = 0.0019 and < 0.0001, respectively), in agreement with the apoptotic cell phenotype observed under the microscope during MTT assays. Therefore, IMD-0354 has a cytotoxitc effect on both human and murine breast cancer cell lines, partially due to induction of apoptosis.

### In vitro effects of combination therapy with IMD-0354 plus chemotherapeutic drugs

**Figure 6:**
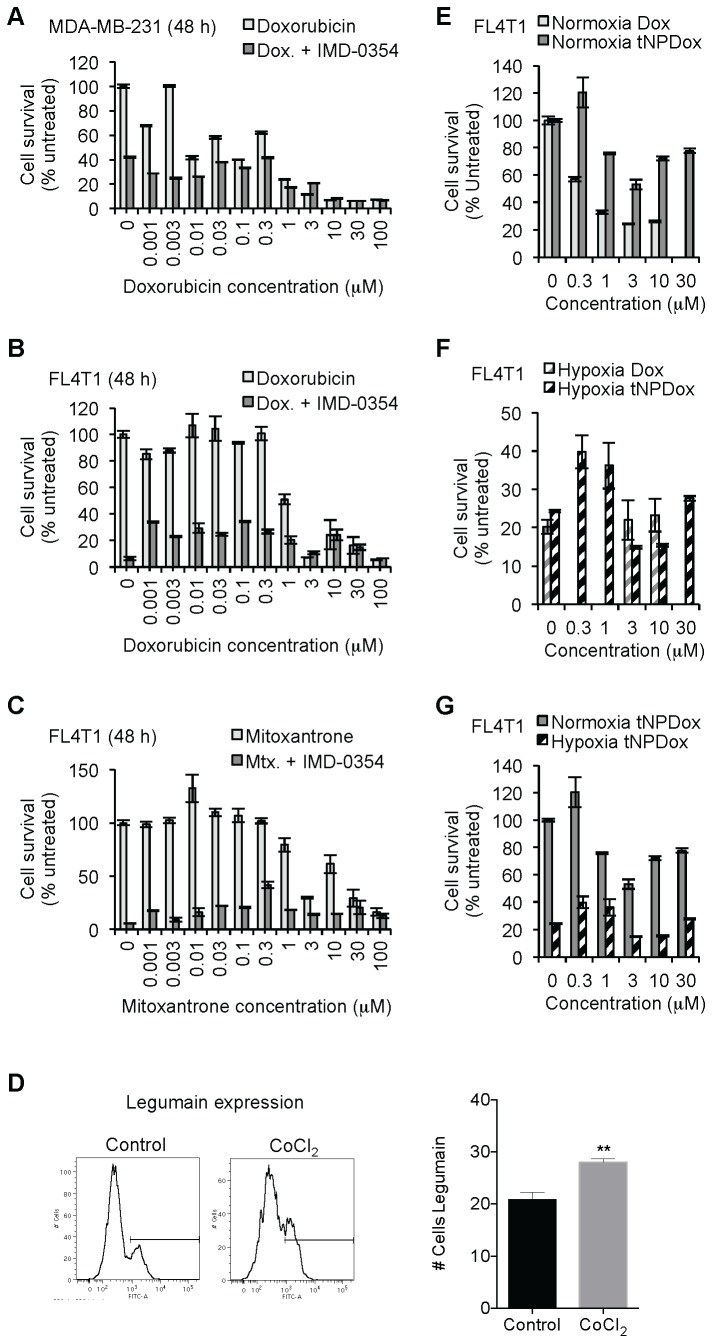
Combination therapy and tNP drug delivery *in vitro*. Viability of MDA-MB-231 cells was assessed by MTT assay after treatment with doxorubicin alone or in combination with IMD-0354 for 48 h (A). Viability of FL4T1 cells was assessed by MTT assay after treatment with doxorubicin (B) or mitoxantrone (C) alone or in combination with IMD-0354 for 48 h. Surface extracellular display of legumain on 4T1 cells assessed by FACS under normoxia (control) and hypoxia (CoCl_2_ treatment). Viability of FL4T1 cells was assessed by MTT assay after treatment with free doxorucibin and tNP-doxorubicin for 48 h under normoxia (E) and hypoxia (F); same data as in E and F, plotted to compare tNP-Dox effect on FL4T1 viability under normoxia and hypoxia. Data are shown as mean ± SEM.

Since CSCs have a higher capability to efflux dyes and drugs, we hypothesized that by targeting CSCs one should also be able to inhibit MDR and increase sensitivity to chemotherapeutic agents. Therefore, we analyzed cell viability by the MTT assay after treatment with a combination therapy of IMD-0354 and chemotherapeutics. Human breast cancer cells MDA-MB-231 were treated with Dox alone or Dox plus 1 µM IMD-0354 (Figure 6A). Although in the micromolar range of Dox (1-100 µM), IMD-0354 was not able to increase its potency (viability ≤ 20%), the NF-κB inhibitor had an additive effect on reducing viable cells together with Dox in the nanomolar range. In fact, at 1-30 nM Dox, viability was reduced from 40–100% to 40-25% by co-treatment with IMD-0354.

Similarly, murine breast cancer cells FL4T1, treated with a combination of IMD-0354 and Dox, resulted in a clear reduction in viability compared to Dox alone, especially at lower concentrations (Figure 6B): at 1-300 nM Dox viability was reduced from 100–80% to 35-30% by co-treatment with IMD-0354. This additive cytotoxic effect of IMD-0354 in FL4T1 cells was not limited to a combination with Dox, but also found in combination with mitoxantrone (Figure 6C): at 1 µM -1 mM of Dox viability was reduced from 90–100% to 20% by co-treatment with IMD-0354.

### In vitro targeted drug delivery

One common detrimental effect of free chemotherapeutic systemic delivery *in vivo* is its toxic side effect on healthy organs. For example, Dox is known for its high cardiac toxicity among off target effects [56]. To overcome this problem, a targeted nanoparticle (tNP) delivery method was previously developed in our laboratory that allows for safe *in vivo* Dox delivery to the tumor microenvironment with little or no toxic side effects [19]. Such nanoparticles specifically target cells expressing legumain in their plasma membranes, which is a common property of the tumor microenvironment, especially under hypoxic conditions [19,23,29,36]. However, before a combination therapy of IMD-0354 plus Dox could be critically tested in animal models, legumain expression and tNP drug delivery had to be assessed *in vitro*.

Legumain expression and translocation to the extracellular portion of the plasmatic membrane can be induced by hypoxia, either by reducing oxygen content or treating cells with 100 µM CoCl_2_ for 24 h. Thus, 4T1 cells were treated with CoCl_2_ and their extracellular, membrane bound legumain analyzed by external immunostaining and FACS (Figure 6D). The treatment mimicking hypoxia with CoCl_2_ induced the externalization of legumain in 4T1 cells (approx. from 20% to 30%; P-value = 0.0015), in agreement with previous work [19].

In fact, targeted delivery of Dox could reduce cell viability *in vitro*. Under normal oxygen conditions (normoxia), tNP loaded with Dox (tNP-Dox) had a lower cytotoxic effect on FL4T1 cells than free Dox (Figure 6E; IC_50_ = 0.20 and 0.06 µM at 48 and 72 h, respectively), probably due to a protective effect of nanoparticles encapsulating Dox. However, under hypoxia (CoCl_2_ treatment), free Dox was unable to further decrease viability (20% viability relative to normoxia) compared to hypoxia treatment itself. Interestingly, tNP-Dox reduced FL4T1 viability even more than free Dox or just hypoxia treatment (from 25% to 15% approx.), probably due to increase of legumain expression and translocation to the extracellular surface (Figure 6F). The improved efficiency of tNP-Dox reducing FL4T1 viability was also observed when its cytotoxic effect was compared between normoxia and hypoxia: viability was reduced from 60–100% to 40-15% (Figure 6G). Together, these results indicate that tNP could facilitate an effective, safe delivery vehicle for Dox or combination therapy treatment.

### IMD-0354 alone or combined with doxorubicin reduce tumor burden in vivo

**Figure 7:**
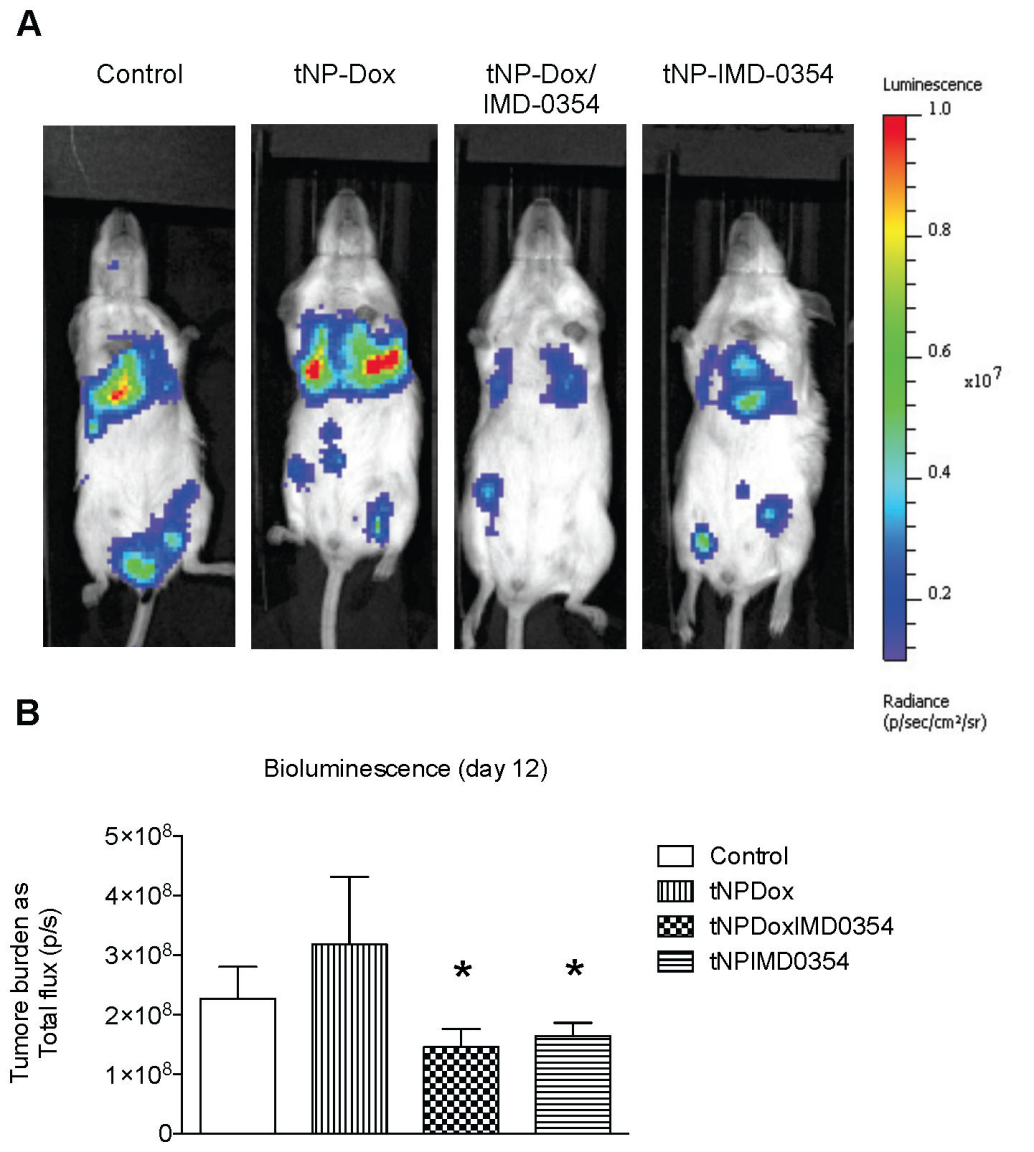
Combination therapy encapsulated into tNPs for drug delivery *in vivo*. Mice bearing experimental metastatic FL4T1 tumors were treated with tNP-Dox, tNP-IMD-0354 or tNP-Dox-IMD-0354. Tumor burden was detected by *in vivo* non-invasive imaging of the firefly luciferase expressing FL4T1 cells after intraperitoneal luciferin injection. Tumor bioluminescence intensity was plotted in pseudocolor over black/white photographs (A) and quantified as total flux in photons/seconds (p/s). Data are shown as mean ± SEM. *P-value < 0.05.

To simulate aggressive or advanced breast cancer in a syngeneic animal model, murine breast cancer cells were chosen for animal experiments. To be able to easily follow tumor burden and metastasis, firefly luciferase expressing 4T1 (FL4T1) cells were used in order to identify tumor cells by non-invasive *in vivo* imaging. Shortly after FL4T1 cells were injected intravenously into female BALBc mice (day 0), treatments with tNP-Dox, tNP-IMD-0354 or tNP-Dox-IMD-0354 were administrated intravenously every 3 days, and tumor cells detected by their bioluminescence. In this experimental mouse metastasis model, FL4T1 cells produced tumors and metastasis in a variety of organs, especially in lungs (Figure 7A; bioluminescence intensity is indicated in pseudocolor as total flux normalized units photons/second). At day 12 of the experiment and after only 6 doses of tNP treatment, a statistical significant reduction in tumor burden (bioluminescence intensity) was achieved by tNP-Dox-IMD-0354 treatment compared to control (Figure 7B; 1.5x10^8^ p/s versus 2.3x10^8^ p/s; P-value = 0.0392). The reduction in tumor burden by tNP containing IMD-0354 proved also statistically significant when compared to tNP-Dox (P-value: 0.0379 and 0.0267, respectively).

## Discussion

Many breast cancer-related deaths are due to MDR and cancer recurrence. The unique capabilities of proliferation, tumorigenesis and chemoresistance of CSCs make these cells an attractive therapeutic target and a good candidate for developing strategies for prevention of cancer recurrence. Here, CSCs were found in both human and murine breast cancer cell lines MDA-MB-231 and 4T1, respectively, by Hoechst 33342 staining of SP. These cells exhibited other stem-like properties typically found in CSCs, such as colony formation in soft agar, tumor-sphere formation in low attachment plates, and expression of Oct4, Nanog, Sox2 and Survivin stemness related genes. In addition, both cell lines were found to have low sensitivity to common chemotherapeutics such as Dox, mitoxantrone and cisplatin. For this reason, both cell lines were chosen as relevant models to study new therapies with potential applicability to treat cancer recurrence.

Since CSCs seem to be a basis for multidrug resistance, minimal residual disease and cancer recurrence, due to their ability to escape from chemotherapeutic cytotoxicity, we hypothesized that drugs targeting CSCs would be an ideal adjuvant for chemotherapeutics to more efficiently treat breast cancer and other solid tumors. However, the broad heterogeneity in CSC markers presents a challenge for successful screening and biomedical application. Therefore, we chose a functional assay based on drug efflux ability common for CSCs of any type of cancer, which is independent of specific ABC transporter expression, genetic variants and species. In this way, we previously identified IMD-0354 as a putative CSC targeting drug. IMD-0354 is a NF-κB inhibitor with anti-inflammatory activity [48,49]. It has also been described to have cytostatic effects on cancer cells, including breast cancer [57]; we, however, are the first to demonstrate its effects on CSCs. In fact, in our functional assay with Hoechst 33342 staining, IMD-0354 was able to reduce SP of CSCs in both human and murine breast cancer cell lines, indicating species independent efficacy. In addition, IMD-0354 was able to reduce or inhibit other CSC specific traits such as colony and tumor-sphere formation and stem-like gene expression. This indicates that IMD-0354 not only inhibits drug efflux, but also reduces the stemness of this subpopulation of cells, a key step necessary to avoid cancer recurrence as proposed in our hypothesis. These results are also in agreement with the recent work by C.T. Yeh and colleagues, where inhibition of the NF-κB pathway decreased CSCs populations in breast cancer [58]. Furthermore, IMD-0354 showed a cytotoxic effect on non-CSCs, the bulk of tumor cells, contributing to the cytotoxic effect of chemotherapeutic drugs on non-CSCs. Actually, IMD-0354 increased the sensitivity to Dox and mitoxantrone, especially at nanomolar concentrations. This synergism suggests that an optimal efficacy of Dox could be achieved at lower concentrations, therefore minimizing chemotherapeutic drug doses as well as the typical cardiac toxicity of Dox and other side effects on patients.

However, there are still issues concerning the high level of Dox toxicity on other healthy organs. To overcome such problems, we previously developed specifically targeted liposomal nanoparticles (tNPs) able to safely deliver Dox specifically to the TME [19]. Since this was proven to be an excellent targeted delivery method for Dox, the same approach was used here for IMD-0354 delivery. The same type of tNPs should also reduce toxic side effects, provide optimal drug concentration, specific drug delivery to the TME and improve pharmacokinetics of a combination therapy. In fact, *in vitro* studies showed that encapsulation of Dox into nanoparticles (tNP-Dox) protects cell viability under normoxia from Dox cytotoxicity as compared to free drug. This indicates that in healthy organs, which are mostly under normoxia, tNP-Dox will not have toxic side effects, especially the severe cardiotoxicity that the free drug produces since Dox remains masked inside the liposomal nanoparticle. However, under hypoxic conditions, tNP-Dox was found more toxic for cells than the free drug. Considering the characteristic hypoxic environment found in solid tumors and their TME [21,23], these tNPs should be able to render chemotherapeutics and adjuvants more effective in the TME, where their cytotoxicity is critically required. Additionally, since tumor cells and tumor macrophages externalize more legumain under hypoxia, these tNPs direct the drugs to the tumor and TME, where they can reach a higher effective dose level without toxicity to healthy organs, as proven earlier for single drug delivery by our group [19].

Furthermore, safety and efficacy of such tNPs was assessed *in vivo* in a murine model of experimental metastasis. In this experiment, mice were intravenously injected with firefly luciferase expressing murine breast tumor cells (FL4T1), which could be detected by their bioluminescence in a non-invasive way. The FL4T1 cells injected in this way rapidly seeded in multiple organs and grew forming metastasis resembling the aggressive progression found in many breast cancer patients. Interestingly, the combination therapy tNP-Dox-IMD-0354 was able to significantly reduce tumor bioluminescence in such a challenging, fast growing metastatic model. Such a decrease in FL4T1 cells accounts for both the number of metastases and tumor size, since bioluminescence is quantified for the whole mouse body. Importantly, this efficacy was achieved without decreasing body weight, one of the first parameters to be affected by acute toxicity. This indication of safe drug delivery is in agreement with more extensive analysis of tNPs safety in mouse models done previously by our group [19].

In summary, improved screening practices and imaging techniques have significantly improved prognosis for women with early stage breast cancer. In contrast, the prognosis and long-term survival of women with metastatic or recurrent disease remains unacceptably poor and claimed over 38,000 lives of American women in 2010. Critically, morbidity as a result of metastatic and recurrent disease is associated with acquired MDR and treatment failure leading to death. CSCs are resistant to conventional chemotherapies and have the capability of proliferation, self-renewal and resistance to hypoxia and other apoptosis triggers, which allow them to repopulate tumors during disease recurrence. Therefore, CSCs remain very attractive targets for molecular therapies to prevent MDR. The innovation of our current work is clearly the combination therapy of an anti-CSC drug, IMD-0354, and a chemotherapeutic drug, Dox, that allow for simultaneous targeting of cancer initiating cells, bulk tumor cells and treatment resistant cells. Importantly, all these effects could be achieved in our targeted delivery system that allows higher doses of chemotherapeutics in the tumor cells and TME and protects healthy organs from the therapeutic agents’ cytotoxic effect. Additionally, drug encapsulation into legumain-directed tNPs synchronizes the pharmacokinetics for the delivery of the different drugs, which has important implications for their clinical application. The potential medical impact of this work is the reduction of breast cancer deaths due to cytotoxic side effects, MDR and tumor progression.
